# A novel F_420_-dependent anti-oxidant mechanism protects *Mycobacterium tuberculosis* against oxidative stress and bactericidal agents

**DOI:** 10.1111/mmi.12127

**Published:** 2012-12-28

**Authors:** Meera Gurumurthy, Martin Rao, Tathagata Mukherjee, Srinivasa P S Rao, Helena I Boshoff, Thomas Dick, Clifton E Barry, Ujjini H Manjunatha

**Affiliations:** 1 Novartis Institute for Tropical Diseases10 Biopolis Road, #05-01, Singapore, 138670, Singapore; 2 Department of Microbiology, Yong Loo Lin School of Medicine, National University of Singapore5 Science Drive 2, Singapore, 117597, Singapore; 3 Tuberculosis Research Section, National Institutes of HealthBldg: 33, 33 North Drive, Bethesda, MD, 20892, USA

## Abstract

*Mycobacterium tuberculosis* (*Mtb*) is an aerobic bacterium that persists intracellularly in host macrophages and has evolved diverse mechanisms to combat and survive oxidative stress. Here we show a novel F_420_-dependent anti-oxidant mechanism that protects *Mtb* against oxidative stress. Inactivation of the *fbiC* gene in *Mtb* results in a cofactor F_420_-deficient mutant that is hypersensitive to oxidative stress and exhibits a reduction in NADH/NAD^+^ ratios upon treatment with menadione. In agreement with the recent hypothesis on oxidative stress being an important component of the pathway resulting in cell death by bactericidal agents, F_420_^−^ mutants are hypersensitive to mycobactericidal agents such as isoniazid, moxifloxacin and clofazimine that elevate oxidative stress. The *Mtb* deazaflavin-dependent nitroreductase (Ddn) and its two homologues Rv1261c and Rv1558 encode for an F_420_H_2_-dependent quinone reductase (Fqr) function leading to dihydroquinones. We hypothesize that Fqr proteins catalyse an F_420_H_2_-specific obligate two-electron reduction of endogenous quinones, thereby competing with the one-electron reduction pathway and preventing the formation of harmful cytotoxic semiquinones, thus protecting mycobacteria against oxidative stress and bactericidal agents. These findings open up an avenue for the inhibition of the F_420_ biosynthesis pathway or Fqr-class proteins as a mechanism to potentiate the action of bactericidal agents.

## Introduction

The aerobic bacterium *Mycobacterium tuberculosis* (*Mtb*) is the causative agent of TB in humans and is responsible for more morbidity than any other bacterial disease. The focal site of TB infection is the lung where *Mtb* infects alveolar macrophages. Upon phagocytosis, the organism resides in membrane-bound vacuoles known as ‘phagosomes’ (Hasan *et al*., 1997). *Mtb* infection in the host is established via a complex interplay between the immune system of the host and survival mechanisms employed by the bacteria, which has evolved diverse mechanisms to combat and survive oxidative and nitrosative stress. A recent study in the non-pathogenic mycobacterial strain *Mycobacterium smegmatis* has suggested a role for FGD1 in combating oxidative stress (Hasan *et al*., 2010). An earlier study, involving whole-genome transposon mutagenesis in *Mtb*, speculated a role for F_420_ in the protection against nitrosative stress (Darwin *et al*., 2003).

F_420_ is a redox active enzyme cofactor found in a variety of methanogenic Archaea and actinomycetales (Jacobson and Walsh, 1984). This soluble 7, 8-didemethyl-8-hydroxy-5-deazariboflavin with a ribosyl-phospholactyl moiety and polyglutamate chain derives its name from the intrinsic 420 nm absorption of its deazaflavin catalytic core FO. FO is the biosynthetic precursor of F_420_ without glutamate residues. F_420_-dependent enzymes are involved in various processes such as methanogenesis, oxygen detoxification, sulphite reduction, antibiotic synthesis and DNA repair in other non-methanogenic archaea and in some actinobacteria (Walsh, 1986; Seedorf *et al*., 2004). A variety of mycobacterial species, including *Mycobacterium leprae* whose genome has undergone substantial reductive evolution (Cole *et al*., 2001), contain both F_420_ and a unique F_420_-dependent glucose 6-phosphate dehydrogenase (G6PD), FGD1. Interest in F_420_ in *Mtb* has accelerated since the discovery of its involvement in the activation of bicyclic 4-nitroimidazole pro-drugs such as PA-824 and Delamanid (OPC-67683) that are currently in a phase II clinical development for tuberculosis (TB) treatment (Stover *et al*., 2000; Matsumoto *et al*., 2006). F_420_H_2_ is utilized by a deazaflavin-dependent nitroreductase (Ddn), in the bioactivation of bicyclic 4-nitroimidazoles (Manjunatha *et al*., 2006; Matsumoto *et al*., 2006). A recent bioinformatics study involving phylogenetic profiling of several bacterial and archaeal genomes based on F_420_ biosynthesis, nominated three dominant families as F_420_-dependent enzymes, one of which was the Ddn family (Selengut and Haft, 2010). Enzymes of the Ddn family are found to be restricted to F_420_ producing bacteria alone. A Ddn homologue in *M. smegmatis* utilizes F_420_H_2_ in the reduction of aflatoxins, a class of fungal xenobiotics (Graham, 2010). Ddn and its homologues therefore form a class of previously uncharacterized F_420_H_2_-dependent reductases with no identified mycobacterial substrate. In *Mtb*, the physiological significance and intracellular role of Ddn and cofactor F_420_ are unclear.

Here we demonstrate the *Mtb* F_420_-deficient mutant is hypersensitive to oxidative stress and to bactericidal agents, supporting the recent hypothesis on oxidative stress being an important component of the pathway leading to bacterial cell death by bactericidal agents (Kohanski *et al*., 2007). Further, for the first time we show that *Mtb* Ddn and its two homologues Rv1261c and Rv1558 encode for F_420_H_2_-dependent quinone reductase (Fqr) function, catalysing the formation of dihydroquinones. We therefore propose a novel F_420_-dependent anti-oxidant mechanism that protects *Mtb* against oxidative stress where Fqr enzymes catalyse an F_420_H_2_-specific obligate two-electron reduction of endogenous quinones and thereby prevent the formation of cytotoxic semiquinones. The findings of this article enhance our current understanding of the complex mechanisms by which *Mtb* combats oxidative stress.

## Results

### Mtb H37Rv *fbiC* knockout mutant is deficient for the production of F_420_

**Fig. 1:**
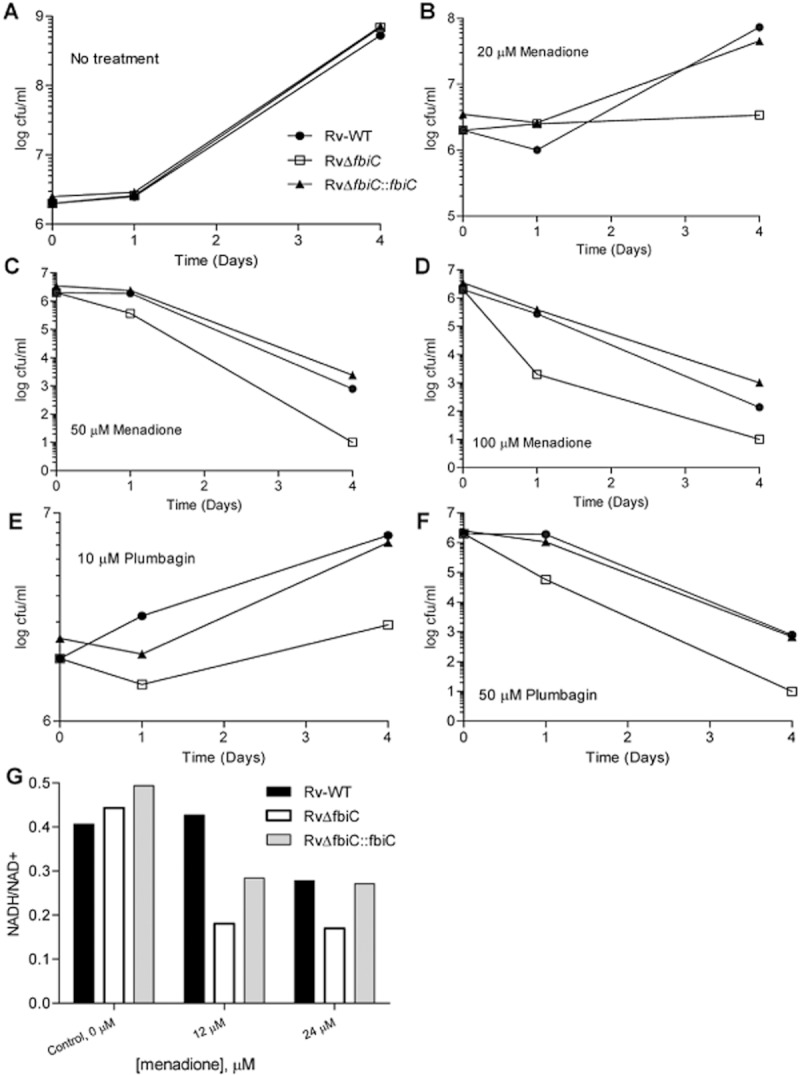
*Mtb* F_420_^−^ mutants are hypersensitive to oxidative stress. A–F. Time-course kill-kinetics of H37Rv WT, H37RvΔ*fbiC* and H37RvΔ*fbiC*::*fbiC* in the absence (A) or presence of 20–100 μM menadione (B–D) or 10–50 μM plumbagin (E and F). G. NADH/NAD^+^ ratios of all three strains when treated with sub-inhibitory concentration of menadione. Colony-forming unit (cfu) data are shown as means of duplicate values from a single biological representative experiment. Experiment was repeated three times, out of which one is represented.

The *fbiC* gene (Rv1173) encodes an 856-amino-acid polypeptide FO synthase that is responsible for the condensation of pyrimidinedione with hydroxyphenyl pyruvate, the first committed step in the F_420_ biosynthetic pathway (Choi *et al*., 2002). An H37RvΔ*fbiC* knockout mutant was generated by homologous recombination and the genotype of the knockout strain was confirmed by PCR and Southern blotting (Fig. S1A–C). The H37RvΔ*fbiC* strain was complemented with pMV306::*fbiC* wherein a single functional copy of the *fbiC* gene was expressed under its native promoter using an integrative plasmid. The phenotype of H37RvΔ*fbiC* and its complemented strain were confirmed by measuring F_420_ fluorescence intensity (λ_ex/em_ 400/470 nm) of crude cell extracts and sensitivity to bicyclic nitroimidazoles (Fig. S1D and E). Lack of F_420_ had no significant effect on *Mtb* growth under normal aerobic conditions in 7H9 medium (Fig. 1A).

### F_420_^−^ mutant is hypersensitive to oxidative stress

Intracellular G6P levels in mycobacteria were found to be ∼ 17- to 130-fold higher than in other bacteria and an *M. smegmatis* mutant deficient in the F_420_-dependent G6PD, FGD1, was hypersensitive to menadione and plumbagin induced oxidative stress (Hasan *et al*., 2010). In order to assess the role of F_420_ and FGD1 in *Mtb*, we evaluated the fitness of the *Mtb* H37RvΔ*fbiC* strain under oxidative stress conditions generated by redox cycling agents such as menadione or plumbagin. It was evident from the results that the H37RvΔ*fbiC* strain was hypersensitive (∼ 1–2 log reduction) to both menadione and plumbagin (Fig. 1B–F). At higher concentrations of the redox cycling agents, the knockout strain displayed a strong growth defect phenotype (1.5–3 log reduction) as early as 24 h post treatment (Fig. 1D and F). In all cases however, the complemented strain was able to restore survival similar to wild-type (WT) levels. Menadione treatment is known to cause significant decrease in NADH/NAD^+^ ratios owing to NADH-dependent quinone reduction (Boshoff *et al*., 2004). Treatment of the H37RvΔ*fbiC* strain with lower concentrations of menadione (10–20 μM) resulted in a significant drop in the NADH/NAD^+^ ratio compared with the WT and complemented strain (Fig. 1G). Similar hypersensitivity and changes in NADH/NAD^+^ ratios was observed in an *M. bovis* BCGΔ*fbiC* mutant and its complemented strain. These data suggest that F_420_H_2_ may compete with NADH in the reduction of menadione. In the absence of cofactor F_420_, mycobacteria therefore exhibit lower NADH/NAD^+^ ratios and become more sensitive to redox cycling agents such as menadione and plumbagin. These observations led us to hypothesize the presence of an F_420_H_2_-dependent quinone reductase in mycobacteria.

### Ddn catalyses F_420_H_2_-dependent reduction of quinone to quinol

**Fig. 2:**
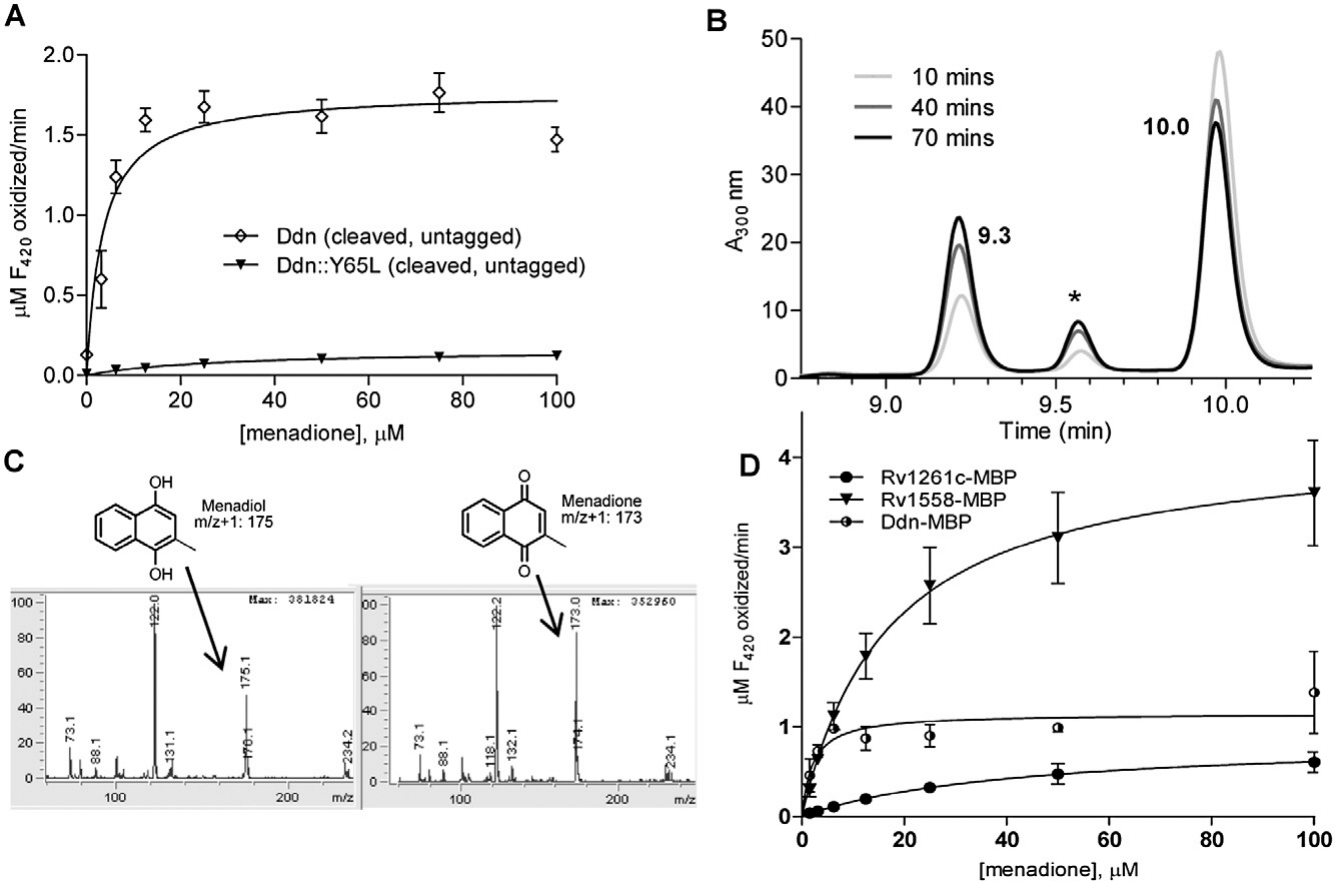
F_420_H_2_-dependent Ddn menadione reductase activity. A. Michaelis–Menten plot for menadione reduction with 100 nM Ddn and inactive Ddn mutant Ddn::Y65L. B. Ddn mediated menadione reduction monitored by LC-MS. A_300_ nm chromatogram peaks for menadione (10 min), menadiol (9.3 min) and an unknown peak (*) are indicated. C. MS profiles for menadiol (9.3 min) and menadione (10 min). D. Michaelis–Menten plot of menadione reduction by Ddn and its *Mtb* homologues, Rv1261c and Rv1558, all are N-terminal MBP-tagged proteins.

A recent study in *M. smegmatis* highlighted the involvement of the FGD1-F_420_ system in the reduction of redox cycling agents (Guerra-Lopez *et al*., 2007; Hasan *et al*., 2010). While these genetic studies in *M. smegmatis* identified a role for FGD1 (through F_420_H_2_) in the quinone reduction, incubation of these substrates with F_420_H_2_ in isolation failed to result in their reduction (Hasan *et al*., 2010) indicating the possible involvement of an F_420_H_2_-dependent enzyme. In *Mtb*, the only enzyme that is known to oxidize F_420_H_2_ is a deazaflavin-dependent nitroreductase (Ddn). Ddn's unambiguous role in the activation of bicyclic 4-nitroimidazoles is well understood; however, its physiological role in *Mtb* is unknown. In order to gain insight into the physiological role of Ddn and its possible role in protecting *Mtb* against oxidative stress, we tested various quinone analogues (Fig. S2) as substrates in a Ddn-mediated F_420_H_2_ oxidation assay. Ddn's quinone reductase activity was initially evaluated with menadione and plumbagin as substrates by monitoring F_420_H_2_ oxidation spectrophotometrically. Absorbance spectra (340–460 nm) with varying concentrations of the quinone analogues as substrates were monitored, with F_420_H_2_ oxidation found to be dependent upon time, substrate concentration and Ddn enzyme (Fig. S3A and B). Steady-state kinetic parameters for Ddn with menadione were determined from a Michaelis–Menten plot (Figs 2A and S3C). Ddn catalysed the reduction of menadione with an apparent *K*_m_ of 3.4 μM and *V*_max_ of 1.8 μM min^−1^ with a *k*_cat_/*K*_m_ of 5.16 min^−1^ μM^−1^ that was 30-fold higher than the non-physiological substrate PA-824 (*k*_cat_/*K*_m_ 0.16 min^−1^ μM^−1^) (Table 1). Moreover, Ddn was specific for F_420_H_2_ in catalysing reduction of quinone substrates and showed no activity when the cofactor was replaced with NADH or NADPH. Based on the recently published high-resolution co-crystal structure of Ddn with cofactor F_420,_ a tyrosine at residue 65 was implicated to play a critical role in stabilizing the Ddn–F_420_ complex, orienting the *Re-*face of the cofactor towards its substrate (Cellitti *et al*., 2012). In order to verify the F_420_H_2_-specific nature of Ddn's quinone reductase activity, we tested the Ddn::Y65L mutant in the menadione reduction assay. The Y65L point mutant of Ddn was not able to utilize F_420_H_2_ to reduce menadione (Fig. 2A), suggesting the importance of the orientation of F_420_H_2_ in the cofactor-binding pocket for the Ddn's menadione reductase activity.

**Table 1:** Summary of Ddn quinone reductase activity with various substrates

Compound, substrate	Enzyme	*K*_m_ (μM)	*k*_cat_ (min^−1^)	*k*_cat_/*K*_m_ (min^−1^ μM^−1^)
Menadione	Ddn^a^	3.44 ± 1.27	17.7 ± 0.12	5.16
	Ddn::Y65L^a^	28.09 ± 4.80	0.161 ± 0.01	0.006
Plumbagin	Ddn^a^	2.17 ± 2.74	5.03 ± 0.36	2.32
1,4-naphthoquinone		2.23 ± 1.19	17.2 ± 0.28	2.58
Dimethyl-naphthoquinone		2.21 ± 0.98	5.37 ± 0.13	2.43
1,4-benzoquinone		ns	ns	ns
Co-Q_0_		12.35 ± 2.65	1.23 ± 0.27	0.1
Co-Q_1_		10.12 ± 2.23	2.15 ± 0.14	0.21
Co-Q_2_		3.14 ± 0.97	5.43 ± 0.12	1.73
PA-824		28.6 ± 3.55	4.7 ± 0.24	0.16
Fluorometry assay for F_420_H_2_ oxidation				
Menadione	MBP-Ddn	2.09 ± 0.91	31.9 ± 2.72	15.3
	MBP-Rv1558	17.29 ± 11.63	98.05 ± 22.95	5.67
	MBP-Rv1261c	47.14 ± 9.87	18.7 ± 1.85	0.40

a Ddn and Ddn::Y65L used were untagged proteins produced by proteolytic removal of MBP.

Experimental data are shown as means ± standard deviation from three independent experiments.

ns, not a substrate.

In order to ensure that F_420_H_2_ oxidation was indeed a result of quinone reduction by the enzyme, we evaluated the quinone reductase activity of Ddn using an alternative readout at A_337_ nM (the absorbance maxima for oxidized menadione) (Hasan *et al*., 2010). A time, enzyme and menadione concentration-dependent decrease in absorbance at 337 nm was observed (Fig. S3D) indicating a depletion of oxidized menadione in the reaction mixtures. To confirm this, the reaction products of menadione reduction were analysed via LC-MS at 10, 40 and 70 min post enzyme addition (Fig. 2B). Chromatography profiles showed a time-dependent increase in the product peak for menadiol (retention time 9.3 min) and concomitant decrease in substrate peak for menadione (retention time 10 min). Mass spectrometry analysis of these peaks revealed the mass (m/z) for menadione and menadiol as 173 Da and 175 Da respectively (Fig. 2C).

### Substrate specificity of Ddn quinone reductase

Quinones are membrane-bound electron carriers of the electron transport chain that structurally vary in the number of isoprene units present in their side-chains. The benzoquinones and naphthoquinones are the two broad categories of quinones present in bacteria. To understand the substrate specificity of Ddn's quinone reductase activity, we analysed enzyme kinetics of Ddn with various naphthoquinone and benzoquinone analogues (Fig. S2). Our data indicated that quinones in general were much better substrates (*k*_cat_/*K*_m_) than PA-824 for Ddn-dependent F_420_H_2_ oxidation. Among the naphthoquinones, plumbagin, DMN (2, 3-dimethyl-naphthoquinone) and NQ (1, 4-napthoquinone) displayed similar activities (*k*_cat_/*K*_m_ ∼ 2 min^−1^ μM^−1^), but were overall inferior to menadione as substrates for Ddn (Table 1). Among the benzoquinone analogues, coenzyme Q_0_ (*k*_cat_/*K*_m_ 0.1 min^−1^ μM^−1^) and CoQ_1_ (0.21 min^−1^ μM^−1^) were poor substrates of Ddn with activities comparable to that of PA-824, whereas coenzyme Q_2_ with two isoprenoid moieties in its side-chain showed improved activity (*k*_cat_/*K*_m_ 1.73 min^−1^ μM^−1^). These results suggest that the isoprenoid hydrophobic tail of the quinone substrate possibly dictates the nature of interaction with Ddn and contributes towards making the quinone analogue a better substrate. In general, naphthoquinones were better substrates of Ddn than benzoquinones, consistent with the fact that the characteristic quinone in mycobacterium is menaquinone, a naphthoquinone with nine isoprenoid moieties in its side-chain. Therefore, one would expect the turnover under physiological conditions to be much higher than what is observed with menadione (kcat 17.7 min^−1^), which requires further investigation. Nevertheless, under *in vitro* enzymatic conditions, Ddn seems to have broad substrate specificity catalysing the reduction of both benzoquinone and naphthoquinone analogues, in addition to bicyclic nitroimidazoles such as PA-824 and OPC-67683.

### Ddn, Rv1261c and Rv1558 form a unique class of F_420_H_2_-specific quinone reductases (Fqr)

Ddn is a member of a large family of proteins that are scattered throughout F_420_-producing actinobacteria (Selengut and Haft, 2010). Interestingly, each of these actinobacterial species is known to have multiple Ddn homologues – *Mycobacterium avium* has 12 homologues, *Mtb* has four homologues and *Nocardia farcinica* has five (Table S2). None of the Ddn homologues have been functionally characterized and they all have very diverse N-terminal sequences (Fig. S4). Homologues in *Mtb*, Rv1261c and Rv1558, with 55–56% amino acid sequence similarity to Ddn, were evaluated for their quinone reductase activity with menadione as the substrate (Fig. 2D). The activity of Rv1588 (*k*_cat_/*K*_m_ 7.24 min^−1^ μM^−1^) was similar to that of Ddn (*k*_cat_/*K*_m_ 9.75 min^−1^ μM^−1^) while that of Rv1261c (*k*_cat_/*K*_m_ 0.46 min^−1^ μM^−1^) was significantly lower (Table 1). Nonetheless, these results established that Ddn and its homologues form a unique class of F_420_H_2_-dependent quinone reductases (Fqr) in *Mtb*.

### F_420_^−^ mutants are defective in a hypoxia-induced dormancy model re-growth assay

**Fig. 3:**
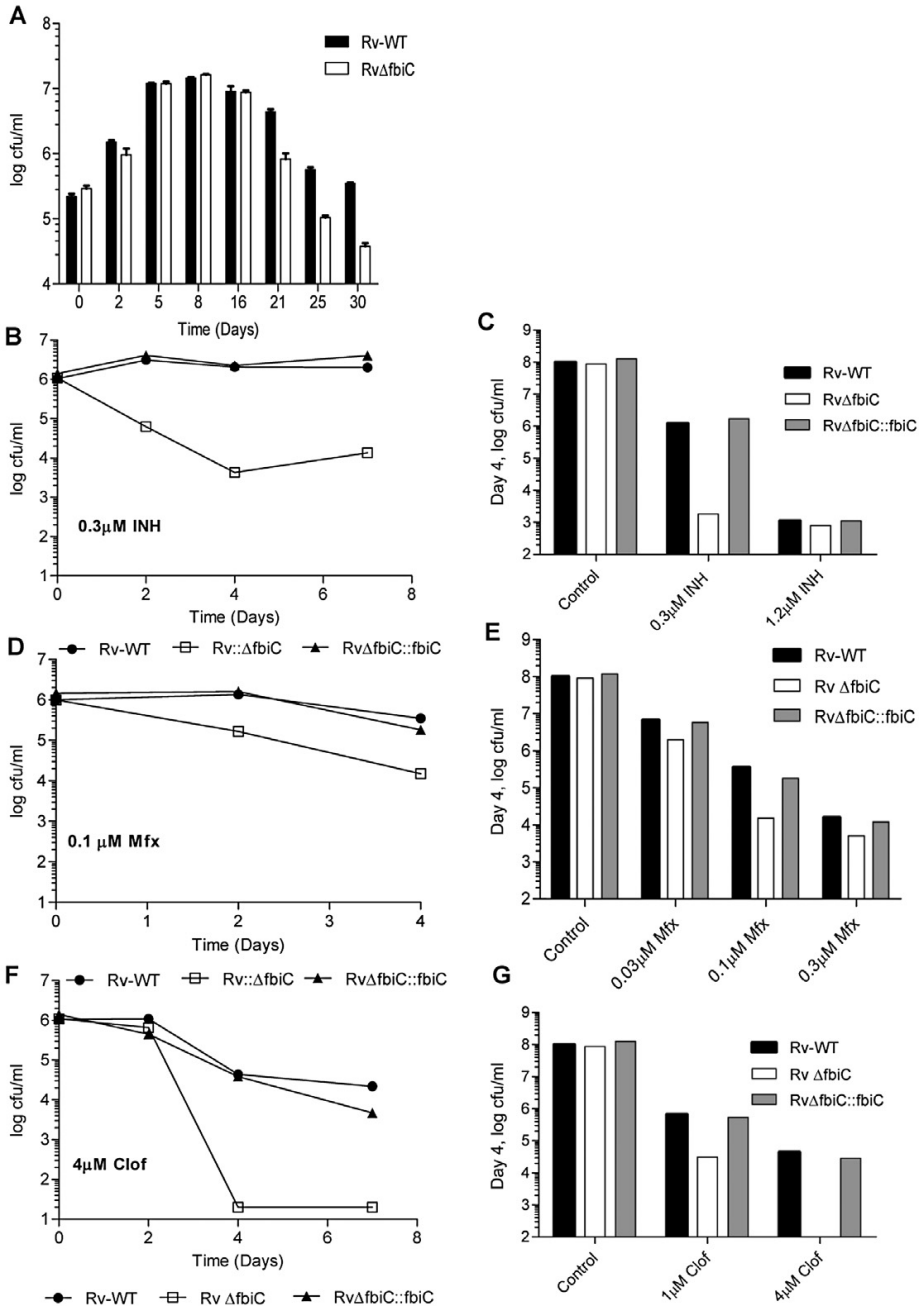
A. *Mtb* F_420_^−^ mutants show survival defect under hypoxia induced dormancy re-growth assay. Growth profiles of Rv-WT and RvΔ*fbiC* strains under the Wayne model for non-replicating persistence. Colony-forming unit (cfu) data are shown as mean ± SD of two independent experiments. B–G. F_420_^−^ mutants are hypersensitive to bactericidal agents. Rv-WT, RvΔ*fbiC* and RvΔ*fbiC*::*fbiC* strains were exposed to indicated concentrations of INH (B and C), moxifloxacin (D and E) and clofazimine (F and G). Viable cells were counted by plating at various time points as indicated (B, D and F) or on day 4 (C, E and G). Colony-forming unit data are shown as means of duplicate values from a single biological representative experiment. Experiment was repeated two times, out of which one is represented.

*Mycobacterium tuberculosis*, albeit an obligate aerobic bacterium, is capable of long-term survival under hypoxic conditions. When exposed to low oxygen tension, *Mtb* ceases replication but maintains viability, a state that the bacillus is known to enter in necrotic granulomas during infection of the lung (Barry III *et al*., 2009). *Mtb* has mechanisms that not only enable entry into hypoxia and survival under these conditions, but also mechanisms that play a role in return to a replicating state upon re-aeration (Rustad *et al*., 2004; Sherrid *et al*., 2010). Upon sudden exposure to oxygen, the bacillus encounters a whole range of reactive oxygen species and combating oxidative stress is expected to be a crucial prerequisite for reactivation of latent TB. In order to evaluate the role of cofactor F_420_ in a physiologically relevant context, the re-growth phenotypes of WT *Mtb* and H37RvΔ*fbiC* on agar plates was monitored after adaptation in a Wayne dormancy model of gradual oxygen depletion (Wayne and Hayes, 1996). The *fbiC* knockout strain showed nearly a log reduction in colony-forming units (cfu) upon entering NRP-2 phenotype (∼ day 21 onwards) (Fig. 3A). Similar growth defect phenotypes were observed for the *M. bovis* BCGΔ*fbiC* strain both in the Wayne model and in the anaerobic rapid shiftdown model both in 7H9 and in Dubos media conditions (Fig. S5). These results suggest a potential role for F_420_ or an F_420_-dependent pathway in re-growth after hypoxia-induced dormancy and therefore possibly in mycobacterial persistence in hypoxic lung lesions and reactivation.

### F_420_^−^ mutants are hypersensitive to bactericidal agents

A recent hypothesis suggests that oxidative stress is a common lethal consequence of bactericidal agents resulting from endogenous formation of superoxide radicals due to increased respiration (Kohanski *et al*., 2007; Belenky and Collins, 2011). Treatment of *M. smegmatis* with bactericidal agents such as ofloxacin or isoniazid (INH) has been shown to result in hydroxyl radical generation (Mukherjee *et al*., 2009). Owing to the hypersensitivity of the F_420_^−^-deficient strain to oxidative stress, we evaluated the effect of bactericidal [INH and moxifloxacin (Mfx)] and bacteriostatic [ρ-aminosalicylic acid (PAS)] agents on growth of H37RvΔ*fbiC* and its complemented strain. We also used the redox cycling TB drug clofazimine which results in increased ROS production by directly interacting with NADH dehydrogenase (NDH2) enzyme (Yano *et al*., 2011). While the complemented strain's growth was comparable to WT, the H37RvΔ*fbiC* strain exhibited a strong growth defect phenotype in the presence of all three cidal agents isoniazid, moxifloxacin and clofazimine (Fig. 3B–G). A similar growth defect to the cidal agents was observed in *M. bovis* BCGΔ*fbiC* (Fig. S6A–C). However, no such phenotype was observed with the bacteriostatic agent PAS in the case of both *M. bovis* BCGΔ*fbiC* and *Mtb* H37RvΔ*fbiC* strains (Figs S6D and S7).

## Discussion

Oxygen serves as the most effective terminal electron acceptor for oxidative phosphorylation in aerobic organisms but is also a source of stress in the form of highly reactive oxygen species such as superoxide (O_2_^−^) and H_2_O_2_ that are formed by the single electron reduction of oxygen (Imlay, 2008). *Mtb*, in addition to being an aerobic bacterium, is an intracellular pathogen that at least initially occupies the phagosomal compartment of host macrophages where it is subject to oxidative and nitrosative stress and has therefore evolved diverse protective mechanisms. An understanding of the defence mechanisms evolved by *Mtb* to cope with oxidative stress is of significant importance in studying the pathogenesis and control of TB. In addition to known protective enzymes such as catalase, superoxide dismutase, peroxidase-peroxynitrite reductase complex and thioredoxin-thioredoxin reductase systems, specialized mechanisms and pathways such as those involved in DNA repair and the proteasome operate to repair the damage caused to macromolecules by oxidative stress (Imlay, 2008; Ehrt and Schnappinger, 2009).

Several lines of evidence (Darwin *et al*., 2003; Hasan *et al*., 2010) have suggested a role for F_420_ and its dependent pathways in protection against oxidative stress. Intracellular G6P levels in several mycobacterial species have shown to be significantly higher when compared with other bacteria (Hasan *et al*., 2010). In addition to classical NADP-dependent G6PDs (ZWF1 and ZWF2), mycobacteria encode an F_420_-dependent G6PD, FGD1 (Purwantini and Daniels, 1996). In most bacteria, one of the two fates of G6P is its oxidation via the pentose phosphate pathway to generate NADPH, an important source of reducing power, which in turn combats various sources of oxidative stress (Ma *et al*., 1998). An *M. smegmatis* mutant deficient in FGD1 is hypersensitive to oxidative stress (Hasan *et al*., 2010), indicating that the presence of an NADPH-dependent G6PD alone to be insufficient, and the lack of FGD1 makes mycobacteria compromised in combating oxidative stress. Results obtained in our study clearly demonstrate that absence of cofactor F_420_ renders *Mtb* hypersensitive to oxidative stress, recapitulating findings obtained in previous studies carried out in *M. smegmatis*.

In addition, here we have demonstrated Ddn's ability to reduce a range of quinone substrates (Table 1) in an F_420_H_2_-dependent manner to their respective dihydroquinone forms. Notably quinones proved to be much better substrates of Ddn than PA-824 and several other bicyclic nitroimidazole substrates evaluated previously (Mukherjee and Boshoff, 2011). Rv1558 and Rv1261c, two homologues of Ddn, were also capable of F_420_H_2_-specific menadione reduction thereby establishing Ddn and its homologues to be a unique class of enzymes in *Mtb*, i.e. the first set of enzymes characterized to possess F_420_H_2_-dependent quinone reductase (Fqr) function in mycobacteria. Experimentally, Ddn has been identified as a membrane protein in *Mtb* (Gu *et al*., 2003; Sinha *et al*., 2005), with an N-terminus likely comprised of an amphipathic helix that has a highly hydrophobic region which may result in peripheral membrane association (Cellitti *et al*., 2012). These data and our current findings of Ddn's ability to reduce quinones point strongly towards Ddn's physiological role as a membrane-associated quinone reductase. In *Mtb*, menaquinone (2-methyl-1,4-napthoquinone moiety with an α-linked chain of on average nine isoprene units) is the sole quinone electron carrier in the respiratory chain (Collins and Jones, 1981). The physiological electron acceptor for *Fqr*-mediated F_420_H_2_ oxidation is therefore likely to be the endogenous menaquinone found in the membrane fraction of *Mtb*.

Quinone reductases in eukaryotes and in bacteria have been well characterized for their roles in protection against oxidative stress (Beyer, 1994; Wang and Maier, 2004; Gonzalez *et al*., 2005; Hong *et al*., 2008). A eukaryotic NAD(P)H quinone reductase, DT-diaphorase (Beyer, 1994; Beyer *et al*., 1997), catalyses the two-electron reduction of quinones, thereby preventing the formation of reactive semiquinones in favour of quinols. Molecular oxygen reacts rapidly with semiquinones to form superoxide (Muller, 1987). A homologue of the eukaryotic quinone reductase in the pathogenic bacteria *Helicobacter pylori* has been shown to be NADPH-dependent and to play an important role in colonization of the host by providing resistance to oxidative stress (Wang and Maier, 2004). ChrR, characterized as a quinone reductase in *Pseudomonas putida* has been observed to combat H_2_O_2_-induced stress by favouring the simultaneous two-electron reduction of quinones (Gonzalez *et al*., 2005). Interestingly, in *Escherichia coli*, which has both ubiquinone (redox potential +113 mV) and menaquinone (redox potential −74 mV), superoxide generation is ascribed mainly to menaquinone likely because of its lower reduction potential (Korshunov and Imlay, 2006) and *Mtb* contains only menaquinone.

**Fig. 4:**
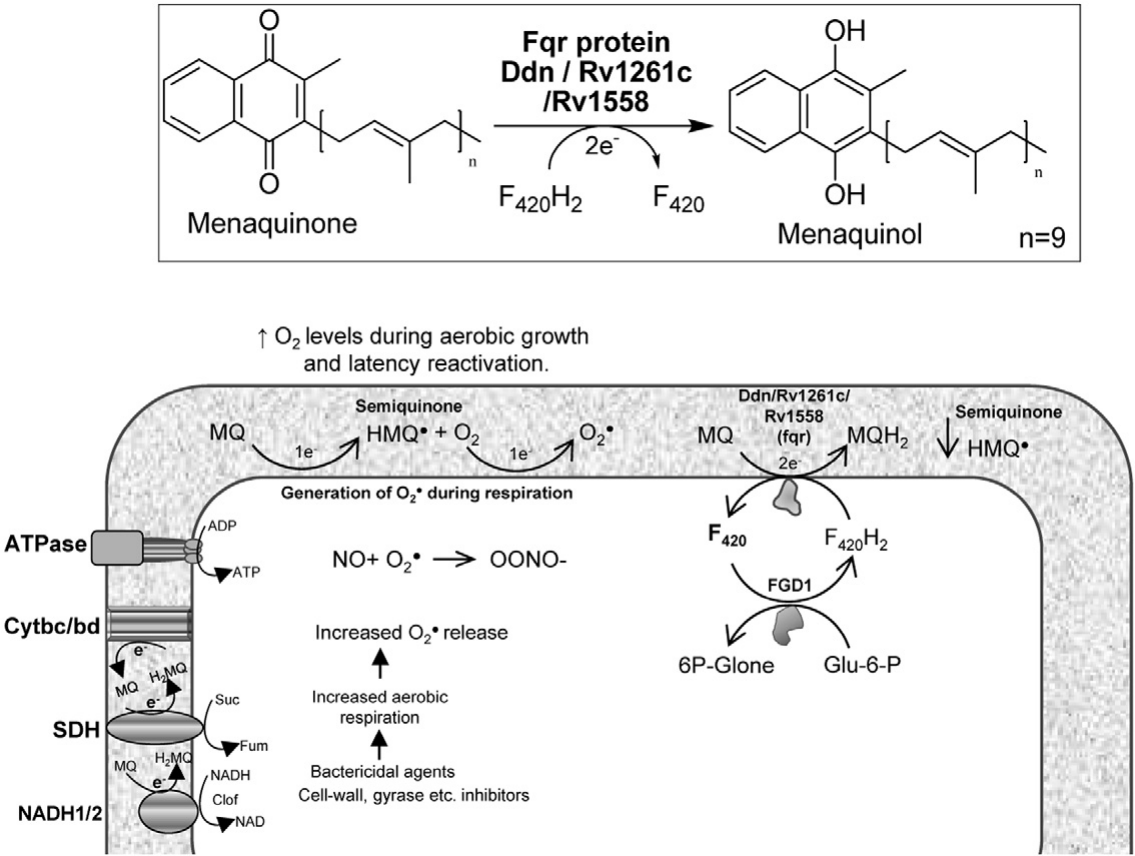
Proposed model for an F_420_-dependent anti-oxidant pathway by Fqr protein family. Fqr proteins catalyse F_420_H_2_-specific obligate two electron reduction of quinones, thereby competing with the harmful one electron reduction pathway as a result of which the formation of cytotoxic semiquinones is avoided. Thus F_420_^−^ as well as FGD^−^ (Hasan *et al*., 2010) strain is hypersensitive to oxidative stress; hypersensitivity of F_420_^−^ strains to nitrosative stress (Darwin *et al*., 2003) is probably due to peroxynitrate formation.

These results lead us to suggest a model in which FGD1 oxidizes G6P to 6-phosphogluconolactone while reducing cofactor F_420_. Fqr proteins (Ddn, Rv1261c and Rv1558) catalyse F_420_H_2_-specific obligate two-electron reduction of quinones to quinol and thereby compete with the harmful one-electron reduction pathway and prevent the formation of cytotoxic semiquinones (Fig. 4). In presence of molecular oxygen, semiquinones lead to the generation of superoxide radical, thus both F_420_^−^ and FGD1^−^ mutants in mycobacteria (Hasan *et al*., 2010) are hypersensitive to oxidative stress. Semiquinone radicals are formed either by the one-electron reduction of quinone or by the one-electron oxidation of quinol. Nevertheless, bioreduction of quinone to quinol achieves potent detoxification as reported here and by several others (Beyer, 1994; Wang and Maier, 2004; Gonzalez *et al*., 2005; Hong *et al*., 2008). The organism therefore appears much more susceptible to bioreductive stress (quinone to semiquinone) than oxidative stress (quinol to semiquinone). This phenomenon further highlights the importance of an obligate two electron reduction system such as F_420_H_2_-Fqr that prevents the accumulation of toxic radicals which can otherwise result in further damage to the organism via oxidative stress.

The G6P-FGD1 and F_420_H_2_-Fqr systems therefore play a crucial role in mechanisms contributing to *Mtb*'s resistance against oxidative stress. Superoxide is known to interact with nitric oxide to form highly reactive peroxynitrite (Beckman *et al*., 1990; Nathan and Shiloh, 2000). As reported earlier (Darwin *et al*., 2003), the *Mtb*Δ*fbiC* mutant displayed a hypersensitive phenotype to nitrosative stress (Fig. S8). This is possibly linked to the increase in oxidative stress and superoxide levels in the mutant, in which case *Mtb*Δ*fbiC* is likely be a prolific generator of a particularly destructive product, peroxynitrite.

In the case of DdnΔN29, an N-terminus truncation mutant whose structure has been solved, the menadione reductase activity was lost; similar to what was observed with PA-824 as a substrate (Cellitti *et al*., 2012). Nevertheless, this mutant protein retained F_420_ binding, indicating the importance of the N-terminus for full enzymatic function and its involvement in substrate binding. Ddn is a member of a large family of proteins distributed exclusively in F_420_-producing actinobacteria (Selengut and Haft, 2010) and multiple Ddn homologues are present in these organisms (Table S2) with diverse N-terminal sequences (Fig. S4). Importantly, the variability of the N-terminus among these homologues suggests a role for this region in defining specificity of cellular functions and substrate specificity. The bioreductive activation of bicyclic nitroimidazoles (PA824, OPC67683 etc.) (Gurumurthy *et al*., 2012), reduction of menadione by Ddn (this study) and the degradation of aflatoxin by an *M. smegmatis* Ddn homologue (Graham, 2010), all seem to share a common mechanism in which hydride is transferred from the reduced deazaflavin F_420_ to the electron deficient ring systems of the substrates. At a quick glance, the broad substrate specificity of the Fqr enzymes (bicyclic nitroimidazoles, benzoquinones and naphthoquinones) may appear puzzling; however, literature is replete with examples of bacterial nitroreductases, known to reduce xenobiotic nitro compounds, exhibiting quinone reductase activity with endogenous substrates (Ross and Siegel, [Bibr b36]; Roldan *et al*., 2008).

The growth defect phenotype of the *fbiC* mutant in the Wayne model may implicate a role for F_420_ either in anaerobic energy metabolism or in combating oxidative stress encountered during reactivation on the agar plate. In a microarray study (Rustad *et al*., 2004), the *fbiC* gene is significantly upregulated (7.4-fold) in the hypoxic environment (7 days exposure to 0.2% oxygen) in comparison with the aerobic conditions and is independent of DosR regulation, suggesting a potential role of F_420_ biosynthesis in hypoxic conditions or synthesis is generating capacity to survive reoxygenation. Global transcriptional analysis has also identified genes that characterize the adaptive response of *Mtb* upon exposure to oxygen and return to favourable growth conditions. Ddn shows a significant upregulation (4.5-fold) upon 24 h of re-aeration of H37Rv cells grown under hypoxia (0.2% oxygen) for 7 days (Sherrid *et al*., 2010); possibly suggesting a protective role of the Ddn against sudden oxidative burst accompanying re-aeration. During aerosol transmission from active TB patients, *Mtb* is exposed to much higher oxygen concentrations than it would be in cavitating lung granulomas, thus the survival defect of the RvΔ*fbiC* mutant during re-aeration may also have an impact on its transmissibility.

Consistent with earlier reports that oxidative stress represents a central component in the processes of death caused by bactericidal agents lead to cell death, F_420_^−^ mutants were observed to be hypersensitive to mycobactericidal agents such as INH, moxifloxacin and clofazimine (Fig. 3B–G). In a murine mouse model, the combination of PA-824 with moxifloxacin (PA-824-Mfx) or with Pyrazinamide (PA-824-PZA) was shown to be more efficacious than the Mfx-PZA combination. Further, the replacement of rifampicin with PA-824 in the Rifampicin-Mfx-PZA (RMZ) regimen was shown to have significantly improved *in vivo* bactericidal activity (Nuermberger *et al*., 2008). Importantly, the PaMZ (PA-824, Mfx and PZA) regimen has recently proven to be highly efficacious in humans in a phase II clinical trial (Diacon *et al*., 2012), supporting our hypothesis of a synergistic interaction between depletion of F_420_H_2_ (by PA-824) and the action of bactericidal agents (Mfx in this case). In the presence of bicyclic nitroimidazoles such as PA-824, Ddn would sequester cellular F_420_H_2_ for the drug bioactivation due to the excess cellular drug concentration relative to endogenous menaquinone levels, thus rendering mycobacterial cells sensitive to oxidative stress caused by other bactericidal agents. Bicyclic nitroimidazoles (PA-824 and OPC67683) in phase II clinical trial possibly operate like a double edged sword as they sequester away cellular F_420_H_2_ and also release intracellular nitric oxide. Further, we would envisage clinical PA-824- or OPC-67683-resistant mutants (FGD^−^ and F_420_^−^) would have greater sensitivity to oxidative stress and to other bactericidal drugs. This supports combination therapies of these drugs and PA-824 to lessen resistance.

Taken together, our findings clearly support the hypothesis that Ddn and its homologues catalyse an F_420_H_2_-specific obligate two electron reduction of endogenous quinones. It is possible that the FGD1-F_420_-Fqr system in *Mtb* serves as a virulence factor, inhibition of which could reduce fitness and enhance the activity of mycobactericidal drugs.

## Experimental procedures

### Bacteria, culture conditions, plasmids and primers

The bacteria, plasmids and primers used in this study are described in Table S1. Culture conditions, determination of minimum inhibitory concentration and colony-forming units for *Mtb* H37Rv and *M. bovis* BCG, isolation of genomic DNA and generation of transformants have all been described earlier (Manjunatha *et al*., 2006).

### Chemicals

Naphthaquinone, 2, 3-dimethyl 1, 4-naphthoquinone (DMN), menadione, plumbagin, benzoquinone and coenzyme Q0-2 were all obtained from Sigma-Aldrich. PA-824 was synthesized as described (Manjunatha *et al*., 2006). All compounds were dissolved in 90% DMSO as 10 mM or 50 mM stocks. F_420_ was purified from *M. smegmatis* mc^2^155 cells as described earlier (Gurumurthy *et al*., 2012).

### Cloning, expression and purification of recombinant WT and mutant Ddn proteins

The coding sequence for Ddn (Rv3547) was amplified, cloned into a Gateway expression system, expressed and purified as an MBP-His_6_-tagged protein as described earlier (Gurumurthy *et al*., 2012). Cleaved, untagged Ddn was used for all activity assays. A gene encoding Ddn but harbouring genetic changes that result in mutation of Tyr65 to Lys65 (Ddn::Y65L) was synthesized for optimal codon usage in *E. coli* (Genescript). Cloning, expression and purification strategy for the point mutant Ddn::Y65L was similar to that of the WT *Mtb* Ddn described earlier (Gurumurthy *et al*., 2012). Ddn homologues in *Mtb* (Rv1261c and Rv1588) were all expressed and purified as MBP-tagged proteins as described earlier (Singh *et al*., 2008).

### Enzyme assays

Quinone reductase activity of Ddn was determined spectrophotometrically by monitoring F_420_H_2_ oxidation at A_400_ nm as described earlier (Gurumurthy *et al*., 2012). Briefly, the assay mixture to contained 100 μM F_420_H_2_, 0–100 μM quinone substrate or PA-824 in a Ddn buffer (200 mM Tris-HCl pH 8.0 with 0.01% Triton X-100) and reaction was initiated by adding 100 nM–1 μM Ddn enzyme (proteolytically cleaved untagged purified Ddn enzyme) in a final volume of 100 μl. Control reactions without the enzyme and without substrate were included for each set of experiments. Quinone reductase activity of Ddn was also determined spectrophotometrically by directly monitoring menadione reduction at A_337_ nm (Hasan *et al*., 2010). F_420_H_2_ was prepared as described previously by the FGD1 catalysed reduction of F_420_ (Singh *et al*., 2008). To determine cofactor specificity for the quinone reductase activity of Ddn, F_420_H_2_ in the assay was replaced with NADH or NADPH and the reaction was monitored for NAD(P)H oxidation. The menadione reductase activities of Ddn homologues Rv1558 and Rv1261c were evaluated with purified MBP-tagged recombinant proteins and monitored F_420_-specific fluorescence (λ_ex/em_ 400/470 nm). Quinone reductase activities of Ddn homologues were directly compared with MBP-tagged Ddn protein under similar conditions. Initial velocities of F_420_H_2_ oxidation were plotted against the substrate concentration and analysed using non-linear regression to the Michaelis–Menten equation using GraphPad Prism 5 (GraphPad Software). Kinetic constants *V*_max_, *K*_m_ and *k*_cat_/*K*_m_ for any given reaction were determined from the plotted data.

High-performance liquid chromatography – mass spectrometry (HPLC-MS) analysis of the enzymatic reactions and controls were performed on Agilent 1100 LC-MS instrument using Luna (3 mm) C18(2) column (50 × 2 mm, Phenomenex). One method was used to analyse all reactions. The column was equilibrated with 95% solution A (0.1% Aq. formic acid) for 1 min. The following gradient was used: 95% to 5% solution A from 0 to 11 min; 5% solution A from 11 to 15 min; 5% to 95% solution A from 15 to 17 min; 95% solution A from 17 to 21.5 min, rest solution B (acetonitrile and 0.1% formic acid). Flow rate was maintained at 0.3 ml min^−1^ and MS was recorder for 60–1000 mass range in the positive mode. This method allowed for the separation of PA-824 (10.3 min), menadione (10.0 min) and menadiol (9.3 min).

### Construction of F_420_-deficient mutant

An F_420_-deficient strain in both *M. bovis* BCG and *Mtb* H37Rv was constructed by allelic exchange using the pYUB845 vector, as described in supplementary text.

### Growth sensitivity to oxidative, nitrosative stress and mycobacterial agents

Mid-log-phase cultures of H37Rv, H37RvΔ*fbiC*, H37RvΔ*fbiC*::*fbiC* strains were diluted to 0.02 OD_600_ in 7H9 medium and exposed to varying concentrations of redox cycling agent such as menadione and plumbagin; bactericidal drugs such as INH, Mfx and Clof; bacteriostatic drugs such as PAS and nitrosative stress inducing acidified sodium nitrite (0–3 mM) for a period of 5–7 days. Serial dilutions of the bacteria were plated on 7H11 plates at various time points to determine cfu ml^−1^. For nitrosative stress inducing conditions, bacteria were grown in acidified (pH 5.5) 7H9 medium as a control.

### Sequence analysis

The primary sequences of the genes were obtained from Tuberculist (http://genolist.pasteur.fr/TubercuList). Multiple sequence alignments were carried out using Vector NTI.
