# Supplementary material for: A novel F420-dependent anti-oxidant mechanism protects Mycobacterium tuberculosis against oxidative stress and bactericidal agents
Source: Mol Microbiol. 2012 Dec 28;87(4):744–55. doi: 10.1111/mmi.12127 (PMC3567243; doi:10.1111/mmi.12127)
Supplement: Supplementary file 1 [file mmi0087-0744-SD1.pdf]

Supporting material

A Novel F_{420}-dependent Anti-oxidant Mechanism Protects *Mycobacterium tuberculosis* against Oxidative Stress and Bactericidal Agents

Meera Gurumurthy[*±ε], Martin Rao[*‡], Tathagata Mukherjee[†], Srinivasa P.S. Rao[*], Helena I. Boshoff[†], Thomas Dick[*¶], Clifton E. Barry 3rd[†] and Ujjini H. Manjunatha[*±§]

*Novartis Institute for Tropical Diseases, 10 Biopolis Road, #05-01, Singapore 138670
[†]Tuberculosis Research Section, National Institutes of Health, Bldg: 33, 33 North Drive Bethesda, MD 20892. [±]Department of Microbiology, Yong Loo Lin School of Medicine, National University of Singapore 5 Science Drive 2, Singapore 117597, Singapore.

[§]Corresponding author: Email: manjunatha.ujjini@novartis.com; Phone: 65-6722 2976 Fax: 65-6722 2917

Present address:
[‡]Max Planck Institute for Infection Biology, Berlin, Germany
[¶]Department of Microbiology, NUS, Singapore
[ε]Department of Medicine, NUS, Singapore

Running title:
Cofactor F_{420}-dependent anti-oxidant mechanism in *Mtb*

Key words:
F420, *Mtb*, Mycobacterium, TB, oxidative stress, quinone reductase

Supplementary Text

Construction of F_{420} deficient mutant. An F_{420} deficient strain in both *M.bovis* BCG and Mtb H37Rv was constructed by allelic exchange using the pYUB845 vector (Bardarov *et al.*, 2002). Briefly, the flanking fragments of the Mtb *fbiC* gene were amplified from mycobacterial genomic DNA by PCR using specific primers (Table S1) and cloned into pYUB854 to flank a hygromycin resistance cassette. *Mtb* H37Rv was transformed using electroporation and transformants (H37RvΔ*fbiC*) were selected on 7H11 plates with 75 μg/mL hygromycin. To generate a complement strain, the H37RvΔ*fbiC* mutant was transformed with an integrative promoter pMV306 (Stover *et al.*, 1991) containing the *fbiC* gene and a 953bp DNA sequence upstream of the gene that encompasses its promoter. Transformants were selected on 7H11 plates containing 75 μg/mL hygromycin and 25μg/mL kanamycin. PCR analysis of the genomic DNA from the WT, H37RvΔ*fbiC*, H37RvΔ*fbiC*::*fbiC* strains using primers (Table S1) to amplify *fbiC*, hygromycin resistance cassette and 5' upstream/ 3' downstream of hygromycin resistance cassette was used to confirm knock out and complementation of the *fbiC* gene. Cofactor F_{420} levels in H37Rv WT, H37RvΔ*fbiC* and H37RvΔ*fbiC*::*fbiC* were determined by analyzing crude cell extracts of the different strains for F_{420} specific fluorescence ($\lambda_{ex/em}$ 400/470 nm) as described previously (Guerra-Lopez *et al.*, 2007). Drug sensitivity profiles of H37Rv WT, H37RvΔ*fbiC* and H37RvΔ*fbiC*::*fbiC* were evaluated by MIC_{99} values of PA-824 for the three strains. MIC_{99} values of INH and rifampicin were determined as controls.

Supplementary Table S1. List of strains, plasmids and primers used in this study

Strain	Description	Source
E.coli TOP 10	Used for routine molecular biology work	Invitrogen
E. coli BL21 (DE3)	Strain used for T7 expression constructs	Invitrogen
M. tuberculosis H37Rv	ATCC # 27294	Lab strain
H37RvΔ*fbiC*	*fbiC* knockout in H37Rv; mutant incapable of synthesizing cofactor F_{420}	This study
H37RvΔ*fbiC*::*fbiC*	H37RvΔ*fbiC* strain complemented with *fbiC* gene	This study
M. bovis BCG	ATCC#35734	Lab strain
BCGΔ*fbiC*	*fbiC* knockout in *M.bovis* BCG; mutant incapable of synthesizing cofactor F_{420}	This study
BCGΔ*fbiC*::*fbiC*	BCGΔ*fbiC* strain complemented with *fbiC gene* via integration of the pMV306-kan-*fbiC* vector	This study
**Plasmid**		**Source**
pET30-FGD1-His	Kan^{r}; pET30b(+) encoding Mtb FGD	(Manjunatha *et al.*, 2006)
pNAT83-Ddn	Kan^{r}; pNAT83 encoding H37Rv Ddn	(Gurumurthy *et al.*, 2012)
pNAT83-Ddn::Y65L	Kan^{r}; pNAT83 encoding H37Rv Ddn::Y65L	This study
pMAL-c2x-Ddn	Amp^{r}; pMALc2x encoding N-terminal MBP-Ddn protein	(Singh *et al.*, 2008)
pMAL-c2x-Rv1261c	Amp^{r}; pMALc2x encoding N-terminal MBP-Rv1261 protein	This study
pMAL-c2x-Rv1588	Amp^{r}; pMALc2x encoding N-terminal MBP-Rv1588 protein	This study
pYUB854	Hyg^{r}; *E.coli-Mycobacterium* shuttle plasmid vector	(Bardarov *et al.*, 2002)
pGOAL17	Vector with *sacB-lacZ* cassette (secondary selection).	(Parish and Stoker, 2000)
pYUB5'-3'*fbiC-PacI*	With (i) 1kb AflII-XbaI and HindIII-XhoI fragments of the 5' upstream and 3' downstream regions of *fbiC* respectively; 6kb PacI fragment from pGOAL17 containing *sacB* and *lacZ*	This study
pMV306-kan	Kan^{r}; integrates at *att* sites on mycobacterial genome	(Cox *et al.*, 2006)
pMV306-hyg-*fbiC*	Hyg^{r}; with 3.6kb HindIII-XbaI fragment containing *fbiC* coding sequence along with 1kb upstream promoter region	(Manjunatha *et al.*, 2006)
pMV306-kan-*fbiC*	Kan^{r} with 3.6kb HindIII-XbaI fragment containing *fbiC* coding sequence along with 1kb upstream promoter region	This study
**Primer (#)**	**Sequence**	**Source**
5'fbiC FP_AflII (1)	5' gccttaagccgtactgcacccacggttc 3'	This study
5'fbiC RP_XbaI (2)	5' gctctagacctcatccacgttcagcgtg 3'	This study
3'fbiC FP_XhoI (3)	5' gcaagcttatggaggagaccatctcgcggatg 3'	This study
3'fbiC RP_HindIII (4)	5' atctcgagagctgctggcggtggacaacgta 3'	This study
pYUBF (5)	5' ggtctgacgctcagtcgaacgaa 3'	This study
pYUBR (6)	5' agtgaggcacctatctcagcgatc 3'	This study
pMV306 F (7)	5' gataaccgtattaccgcctt 3'	This study
pMV306 R (8)	5' ccagtctttcgactgagcct 3'	This study
hyg –int-FP (9)	5' gagagcctcgcgtcggagtc 3'	This study
hyg –int- RP (10)	5' gcagttgcaccaggctgtag 3'	This study
fbiC- int- FP (11)	5' cgccgaacctggtgtctggcgacgaa 3'	This study
fbiC- int- RP (12)	5' ctcgtcgtccaggatttccgcggcg 3'	This study
fbiC5' RC FP (13)	5' gaagttgatctgcggtgcca 3'	This study
fbiC 3' RC RP (14)	5' gggtgaactacgaacagatca 3'	This study
hygF (15)	5' cacgagcagacctcactagc 3'	This study
hygR2 (16)	5' acggttgctagcacgcgca 3'	This study

Supplementary Table S2. Distribution of FQRs in actinobacteria

Genera	Species (# of homologues)	Homologues*
Mycobacterium	*M. tuberculosis* (4)	Ddn (NP_218064)<br>NP_215777 (Rv1261c)<br>NP_216074 (Rv1558)<br>NP_217694 (Rv3178)
	M. bovis BCG (4)	BCG1320c<br>BCG1610<br>BCG3203<br>BCG3611
	M. smegmatis (5)	YP_886389<br>YP_887170<br>YP_887322<br>YP_889280<br>YP_890224
	M. leprae (0)	-
	M. ulcerans (3)	YP_905531<br>YP_907625<br>YP_907922
	M. avium (7+5)	NP_959060<br>NP_959453<br>NP_959999<br>NP_960197<br>NP_961116<br>NP_961408<br>NP_961613<br><br>YP_879893<br>YP_880486<br>YP_880690<br>YP_882400<br>YP_882624
	M. vanbaalenii (6)	YP_951851<br>YP_952028<br>YP_953430<br>YP_YP955238<br>YP_956038<br>YP_956611
	M. gilvum (5)	YP_001132268<br>YP_001132775<br>YP_001133106<br>YP_001135043<br>YP_001136856
Rhodococcus sp	*R. sp* RHA1 (7)	YP_700478<br>YP_701052<br>YP_703786<br>YP_704097<br>YP_704621<br>YP_705330<br>YP_708857
Nocardia	*N. farcinica* (5)	YP_119555

		YP_118018<br>YP_121065<br>YP_117786<br>YP_119109
	Nocardioides (1)	YP_921489
Salinispora	*S. tropica* CNB (3)	YP_001157939<br>YP_001158209<br>YP_001161017
	S. arenicola (3)	YP_001535881<br>YP_001536213<br>YP_001539390
Streptomyces	*S. coelicolor* (2)	NP_625077<br>NP_627523
	S. avermitilis (1)	NP_828641
Frankia	*Frankia. sp* (8)	YP_001504850<br>YP_001505904<br>YP_001504988<br>YP_001506366<br>YP_001506374<br>YP_001506525<br>YP_001509650<br>YP_480522
Kinetococcus	*K. radiotolerans* (1)	YP_001432596
Saccharopolyspora	*S. erythraea* (3)	YP_001103498<br>P_001105121<br>YP_001105781
Rosiflexus	*R. castenholzii* (1)	YP_001432596
	Rosiflexus sp. (1)	YP_001277041
Chloroflexus	*C. aurantiacus* (1)	YP-001635198
	C. aggregans (1)	ZP_01516957
Janibacter	*Janibacter sp.* (3)	ZP_00994801<br>ZP_00995720<br>ZP_00996801
	Marine.actinobacterium (2)	ZP_01131276<br>ZP_01129278
	Marine.gamma.proteobacterium (1)	ZP_01616173
	Proteobacterium (1)	ZP_01615264

* Gene accession numbers

Reference List

Bardarov,S., Bardarov Jr S Jr, Pavelka Jr,M.S.J., Sambandamurthy,V., Larsen,M., Tufariello,J. *et al.* (2002) Specialized transduction: an efficient method for generating marked and unmarked targeted gene disruptions in *Mycobacterium tuberculosis, M. bovis* BCG and *M. smegmatis*. *Microbiology* **148**: 3007-3017.

Cox,H., Kebede,Y., Allamuratova,S., Ismailov,G., Davletmuratova,Z., Byrnes,G. *et al.* (2006) Tuberculosis recurrence and mortality after successful treatment: impact of drug resistance. *PLoS Med* **3**: e384.

Guerra-Lopez,D., Daniels,L., and Rawat,M. (2007) *Mycobacterium smegmatis mc2 155 fbiC* and *MSMEG_2392* are involved in triphenylmethane dye decolorization and coenzyme F420 biosynthesis. *Microbiology* **153**: 2724-2732.

Gurumurthy,M., Mukherjee,T., Dowd,C.S., Singh,R., Niyomrattanakit,P., Tay,J.A. *et al.* (2012) Substrate specificity of the deazaflavin-dependent nitroreductase from *Mycobacterium tuberculosis* responsible for the bioreductive activation of bicyclic nitroimidazoles. *Febs Journal* **279**: 113-125.

Manjunatha,U.H., Boshoff,H., Dowd,C.S., Zhang,L., Albert,T.J., Norton,J.E. *et al.* (2006) Identification of a nitroimidazo-oxazine-specific protein involved in PA-824 resistance in *Mycobacterium tuberculosis. Proc Natl Acad Sci U S A* **103**: 431-436.

Parish,T., and Stoker,N.G. (2000) Use of a flexible cassette method to generate a double unmarked *Mycobacterium tuberculosis tlyA plcABC* mutant by gene replacement. *Microbiology* **146 (Pt 8)**: 1969-1975.

Singh,R., Manjunatha,U., Boshoff,H.I., Ha,Y.H., Niyomrattanakit,P., Ledwidge,R. *et al.* (2008) PA-824 kills nonreplicating *Mycobacterium tuberculosis* by intracellular NO release. *Science* **322**: 1392-1395.

Stover,C.K., de la Cruz,V.F., Fuerst,T.R., Burlein,J.E., Benson,L.A., Bennett,L.T. *et al.* (1991) New use of BCG for recombinant vaccines. *Nature* **351**: 456-460.

A

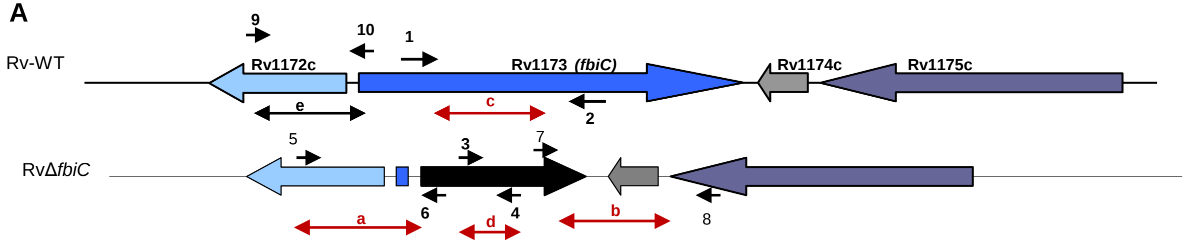

B

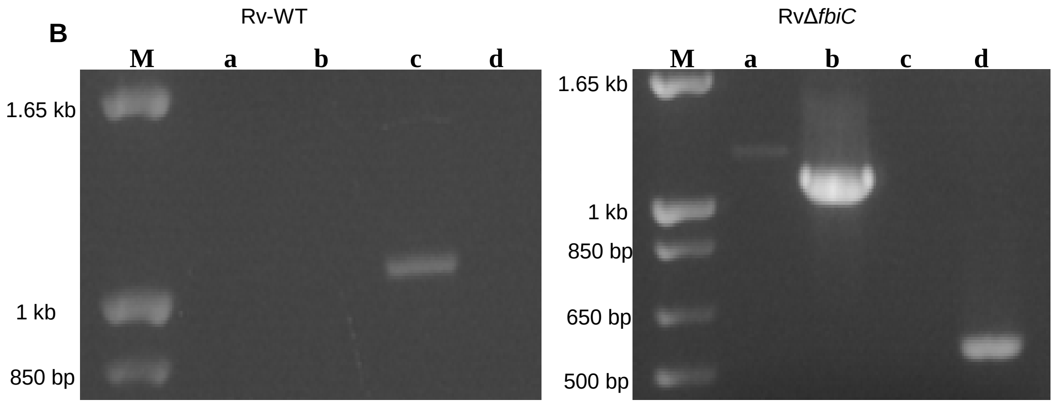

M = 1kb plus DNA ladder (In vitrogen), a and b are 5´ and 3' insertion fragments respectively
c = *fbiC* internal fragment, d = Hygromycin internal fragment

C

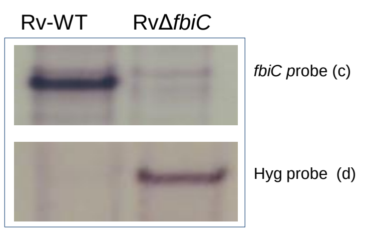

D

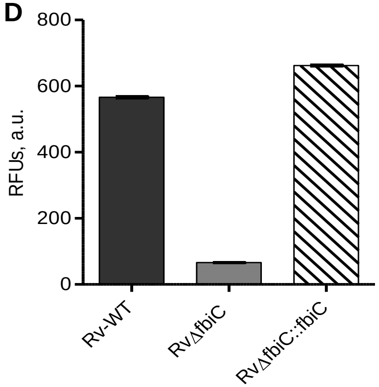

E

	MIC_{99} µM		
	**Rv-WT**	**RvΔ*fbiC***	**RvΔ*fbiC*::*fbiC***
**INH**	0.4	0.4	0.4
**Rif**	<0.01	<0.01	<0.01
**PA-824**	0.8	>100	0.8

Supplementary Figure S1. Generation and characterization of H37Rv *fbiC* deletion strain.
A. *fbiC* locus in the H37Rv WT and H37RvΔ*fbiC* strains. Numbers and alphabets indicate respectively primers and their respective PCR products.
B. PCR confirmation of *fbiC* deletion. **C.** Southern-blotting confirmation of *fbiC* deletion. DIG labeled versions of PCR products **'c'** and **'d'** described in S1A and B were used for Southern blotting. **D.** Cofactor F_{420} levels in the different strains analyzed via specific fluorescence output (Ex/Em 400/470nm) of crude cell extracts.
E. Minimum Inhibitory Concentrations of the anti-TB drugs INH, Rifampicin and PA-824 for the H37Rv WT, *fbiC* knockout and *fbiC* complemented strains.

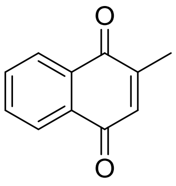

Menadione (2-Methyl-
1,4-naphthoquinone

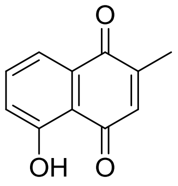

Plumbagin
(5-hydroxy-2-methyl-
1,4-naphthoquinone

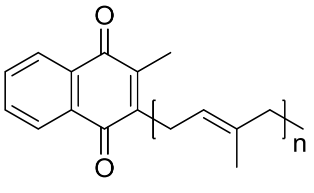

Menaquinone
(Mtb n= 9)

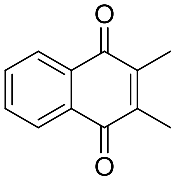

2,3-dimethyl-1,4-
naphthaquinone

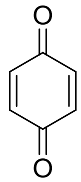

Benzoquinone
(1,4 benzoquinone)

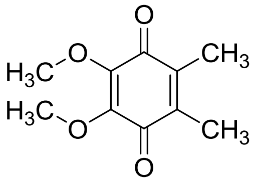

Co-Q_0 (2,3-dimethoxy-5-
methyl-1,4-benzoquinone

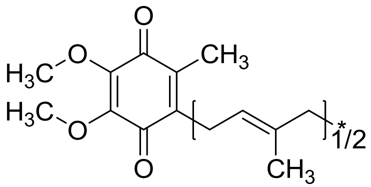

CoQ1/2 (n= 1/2) 2,3-dimethoxy-5-
methyl-6-geranyl-1,4-benzoquinon

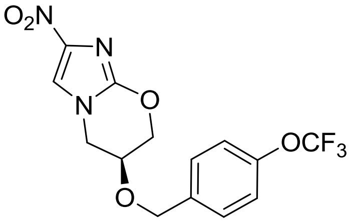

PA-824

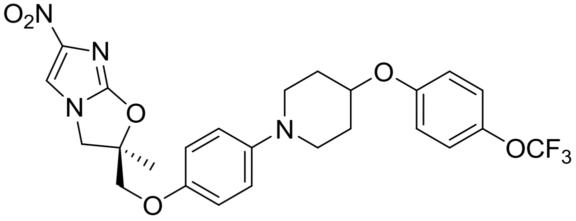

OPC 67683

Supplementary Figure S2. Chemical structures of nitroimidazole and quinone analogs used in this study.

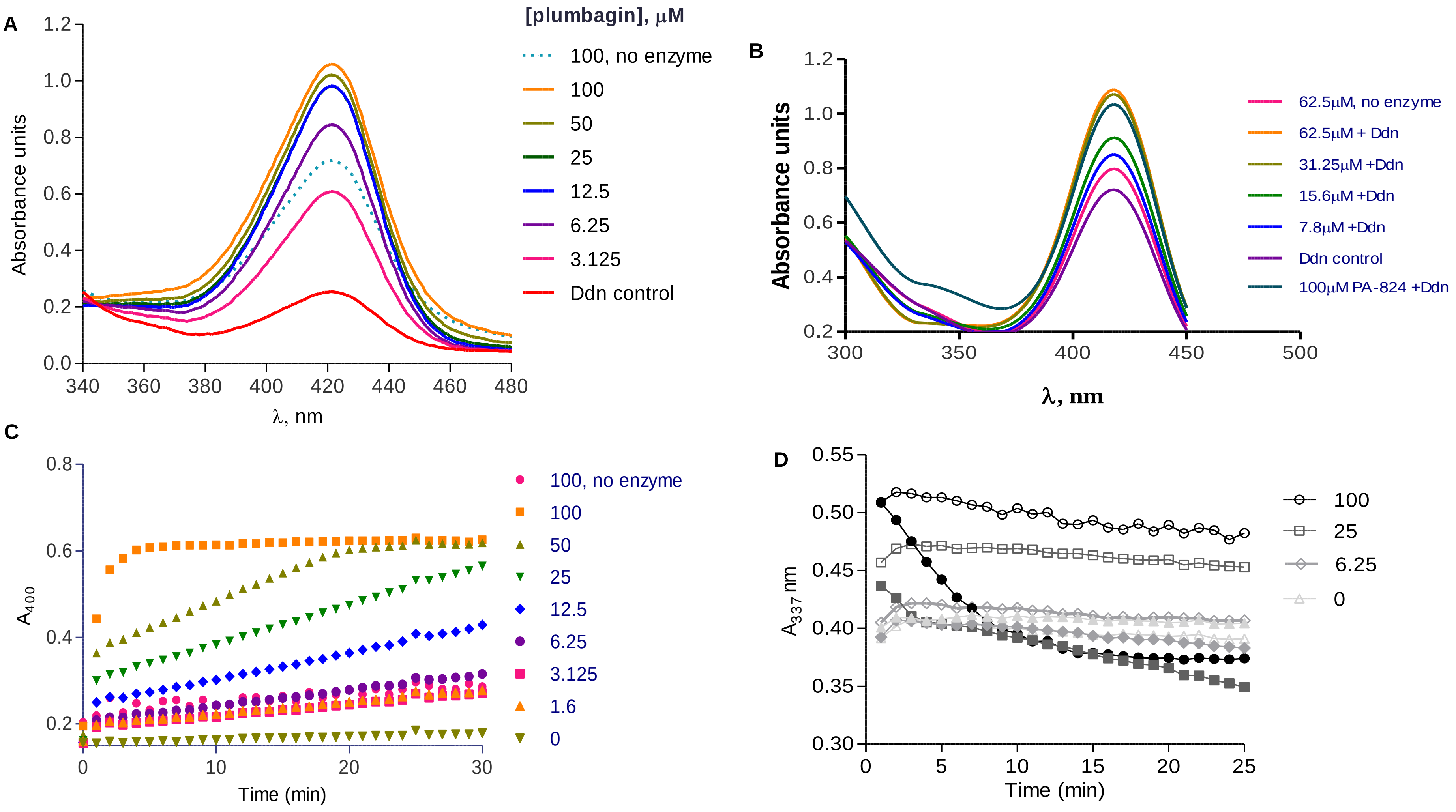

Supplementary Figure S3. $F_{420}H_2$ dependent Ddn quinone reductase activity.
A and B Absorbance spectrum of varying concentrations of plumbagin and menadione respectively with 100 µM of $F_{420}H_2$ after 25 minutes reaction with 1 µM Ddn. **C.** Time course kinetics of menadione reduction by Ddn measured by $F_{420}H_2$ oxidation (A400). **D**. Time course kinetics of menadione reduction by Ddn at A337 (absorption for oxidized menadione). Filled and open symbols represent with and without Ddn enzyme (100 nM) respectively.

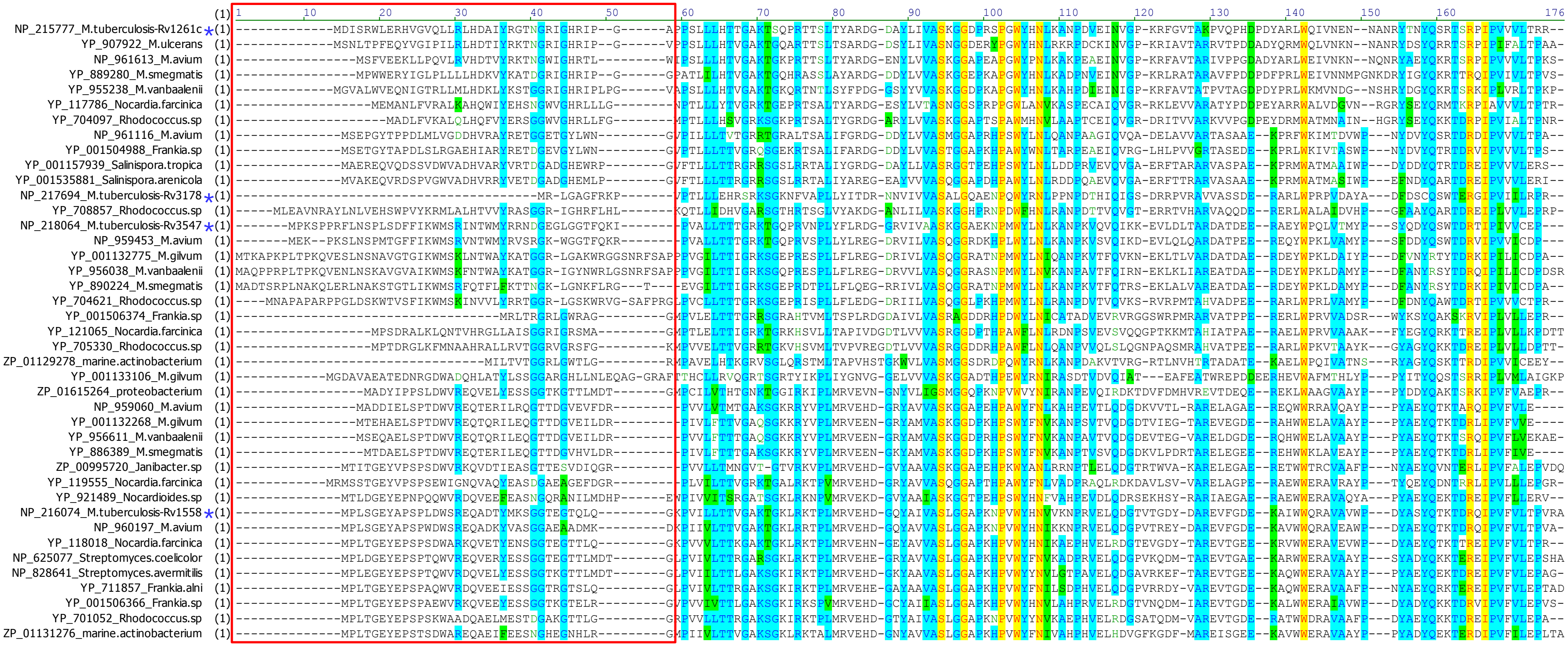

Supplementary Figure S4. Sequence alignment of a few selected Ddn homologues in actinobacteria. Accession number of each protein is cited by the organism name. The four Mtb homologues are marked with an asterisk. Diversity in the N-terminal sequence is highlighted by the red box. Sequence alignment was done by using the Vector NTI-AlignX software.

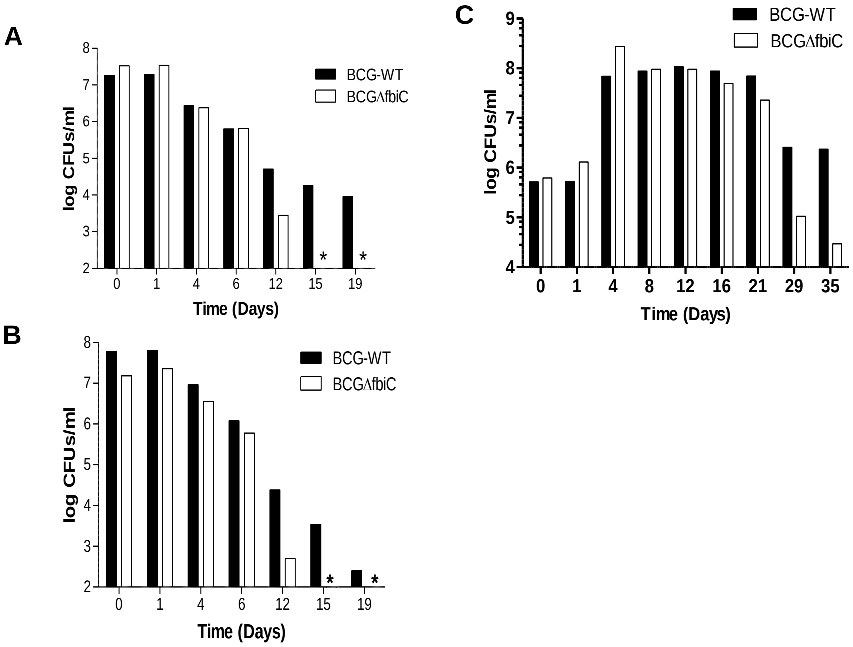

Supplementary Figure S5. BCG F_{420}^{-} mutants show survival defect in hypoxia induced dormancy re-growth assay. WT and Δ*fbiC* BCG strains were subject to growth in a "rapid oxygen depletion model" in either **A.** 7H9 or **B.** Dubos medium; viable cells were counted by plating at various time points. **C.** Growth profiles under the Wayne model for Non-replicating persistence; viable cells were counted by plating at various time points. CFU data are shown as means of duplicate values from a single biological experiment. Experiment was repeated twice, out of which one is represented. Asterisk represents no CFUs detected at the limit of detection of 10 CFUs.

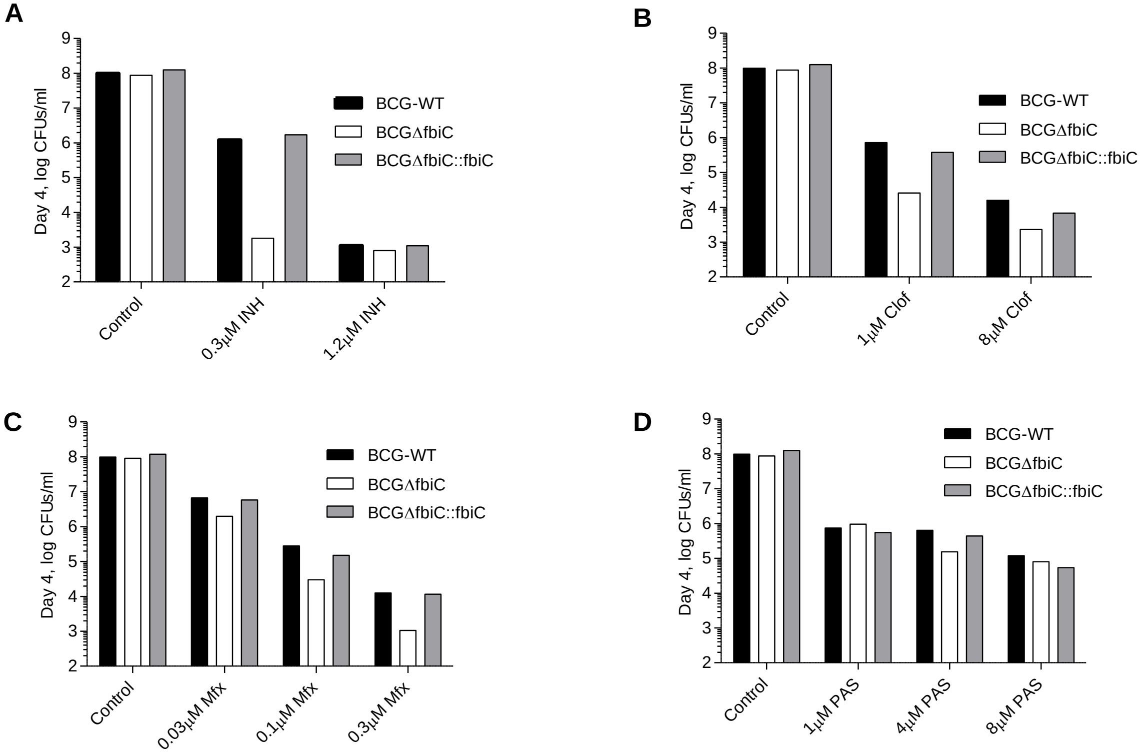

Supplementary Figure S6 *M. bovis* BCG F_{420}^- mutant is hypersensitive to bactericidal agents. BCG WT, ΔfbiC and ΔfbiC::fbiC strains were exposed to indicated concentrations of INH (**A**), Clofazimine (**B**), Moxifloxacin (**C**) and PAS (**D**)for 4 days and viable cells were counted by plating. CFU data are shown as means of duplicate values from a single biological experiment. Experiment was repeated twice, out of which one is represented.

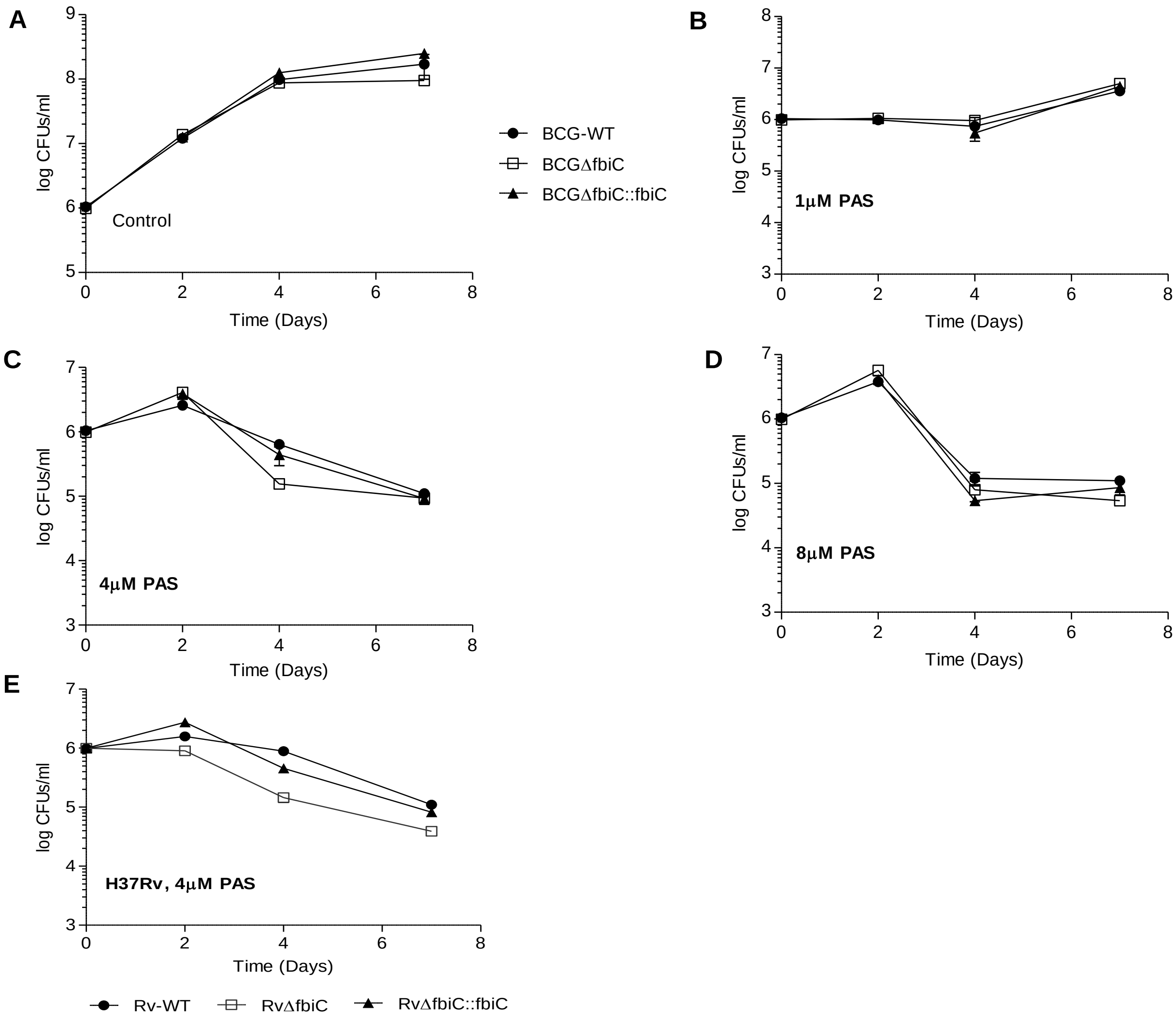

Supplementary Figure S7 *M. bovis* BCG and *Mtb* H37Rv F_{420}^{-} mutants are not hypersensitive to PAS. WT, ΔfbiC and ΔfbiC::fbiC strains of BCG (**A-D**) and H37Rv (**E**) were exposed to indicated concentrations of PAS and viable cells were counted by plating. CFU data are shown as means of duplicate values from a single biological experiment. Experiment was repeated twice, of which one is represented.

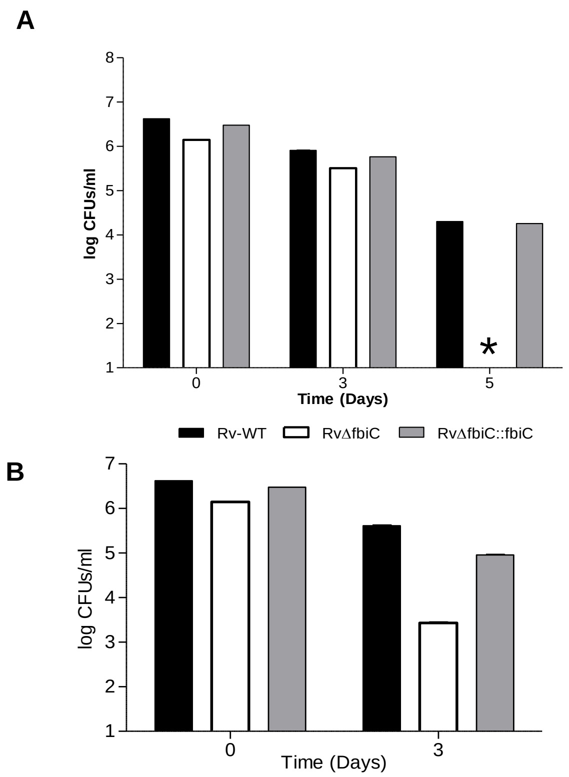

Supplementary Figure S8. F_{420}^{-} mutants show survival defect under nitrosative stress. *Mtb* H37Rv WT, Δ*fbiC* and Δ*fbiC*::*fbiC* strains were grown in 7H9 media with **A.**1.5 mM $NaNO_2$ or **B.** 3.0 mM $NaNO_2$ under acidic conditions (pH 5.5); viable cells were counted by plating at various time points. CFU data are shown as means of duplicate values from a single biological experiment. Experiment was repeated twice, of which one is represented. Asterisk represents no CFUs detected at the limit of detection of 10 CFUs.
